# Detection of *N*-acyl homoserine lactones using a *traI*-*luxCDABE*-based biosensor as a high-throughput screening tool

**DOI:** 10.1186/1472-6750-8-59

**Published:** 2008-07-30

**Authors:** Steve P Bernier, Anne L Beeston, Pamela A Sokol

**Affiliations:** 1 Department of Microbiology and Infectious Diseases, University of Calgary, Calgary, Canada; 2 Département de Microbiologie, Institut Pasteur, Paris, France; 3 Canadian Food Inspection Agency, Lethbridge, Canada

## Abstract

**Background:**

Bacteria use *N*-acyl homoserine lactone (AHL) molecules to regulate the expression of genes in a density-dependent manner. Several biosensors have been developed and engineered to detect the presence of all types of AHLs.

**Results:**

In this study, we describe the usefulness of a *traI-luxCDABE*-based biosensor to quickly detect AHLs from previously characterized mutants of *Burkholderia cenocepacia *and *Pseudomonas aeruginosa *in both liquid and soft-agar co-culture assays in a high-throughput manner. The technique uses a co-culture system where the strain producing the AHLs is grown simultaneously with the reporter strain. Use of this assay in liquid co-culture allows the measurement of AHL activity in real time over growth. We tested this assay with *Burkholderia cenocepacia *and *Pseudomonas aeruginosa *but it should be applicable to a broad range of gram negative species that produce AHLs.

**Conclusion:**

The co-culture assays described enable the detection of AHL production in both *P. aeruginosa *and *B. cenocepacia *and should be applicable to AHL analysis in other bacterial species. The high-throughput adaptation of the liquid co-culture assay could facilitate the screening of large libraries for the identification of mutants or compounds that block the synthesis or activity of AHLs.

## Background

Bacteria possess various regulatory systems that enable them to quickly adapt to subtle changes in their environment. Variation in population density is a condition that bacteria are capable of perceiving and responding to in order to coordinate a myriad of behaviors, often referred as quorum sensing (QS) [1]. The molecular basis of most QS-dependent systems in Gram-negative bacteria is similar to the LuxI/R system of *Vibrio fischeri*, well known for the control of bioluminescence [2]. Briefly, a *luxI *homologue encodes for a *N*-acyl homoserine lactone (AHL) synthase that catalyzes the synthesis of AHL signal molecules, and a *luxR *homologue encodes a sensor/response regulator, which binds its cognate AHL in order to regulate the expression of genes usually in a density-dependent manner [3]. Recognition of cognate AHLs is dependent on the length of the acyl-chain and position C3 which can be either unmodified or carry an oxo- or hydroxyl group substitution.

Several biosensor systems have been developed that vary with respect to sensitivity and specificity for the detection of the AHL signals (reviewed in [4]). Recently, our laboratory developed a new bacterial biosensor using the TraI/R system of *Agrobacterium tumefaciens *to first detect the presence of AHLs in the lungs of rats and mice infected with *Burkholderia cenocepacia *[5] and subsequently from mucopurulent secretions of cystic fibrosis patients infected with *Pseudomonas aeruginosa *and/or *B. cepacia *complex organisms [6]. This biosensor system was engineered in the strain *A. tumefaciens *A136 cured of the Ti plasmid [7] to eliminate the TraI/R QS system. Our reporter strain carries two plasmids, pCF218, which constitutively produces the TraR response regulator [8] and pMV26 [5,6], which contains the *traI *promoter fused to the *luxCDABE *operon [9]. TraR binds AHLs present in the same environment as the reporter strain resulting in an AHL-TraR complex that subsequently binds to a specific sequence within the *traI *promoter on pMV26, triggering the transcription of the *luxCDABE *operon and the production of bioluminescence. This biosensor was shown to respond to AHLs with acyl side chains ranging from 4 to 12 carbons with greater sensitivity to AHLs with longer side chains and those with 3-oxo-substitutions [6].

In the present study, we describe the use of the *traI-luxCDABE *fusion [5,6] as a powerful bacterial biosensor for the *in vitro *detection of AHLs in co-culture assays in both liquid and semi-solid agar and its application as a high-throughput screening tool using *B. cenocepacia *and *P. aeruginosa*, two species with well characterized quorum sensing systems. *B. cenocepacia *has two QS systems, designated CepIR and CciIR that regulate genes involved in virulence, biofilm formation, swarming motility, and regulation [10]. *B. cenocepacia *strain K56-2 produces two AHLs with *N*-octanoyl-l-HSL (C_8_-HSL) synthesized primarily by the *cepI *gene product and *N*-hexanoyl-l-HSL (C_6_-HSL) synthesized mainly via the CciI synthase [11-13]. *P. aeruginosa *has two QS systems, designated LasIR and RhlIR, that influence the expression of approximately 5% of the genome including genes involved in virulence and biofilm formation [14]. *P. aeruginosa *PAO1 produces *N*-oxododecanoyl-l-HSL (3-oxo-C_12_-HSL) and *N*-oxooctanoyl-l-HSL (3-oxo-C_8_-HSL) via the LasI synthase, and *N*-butanoyl-l-HSL (C_4_-HSL) [15] and *N*-hexanoyl-l-HSL (C_6_-HSL) via the RhlI synthase [16].

## Results and discussion

### Detection of AHLs in real time using a liquid co-culture assay

**Figure 1:**
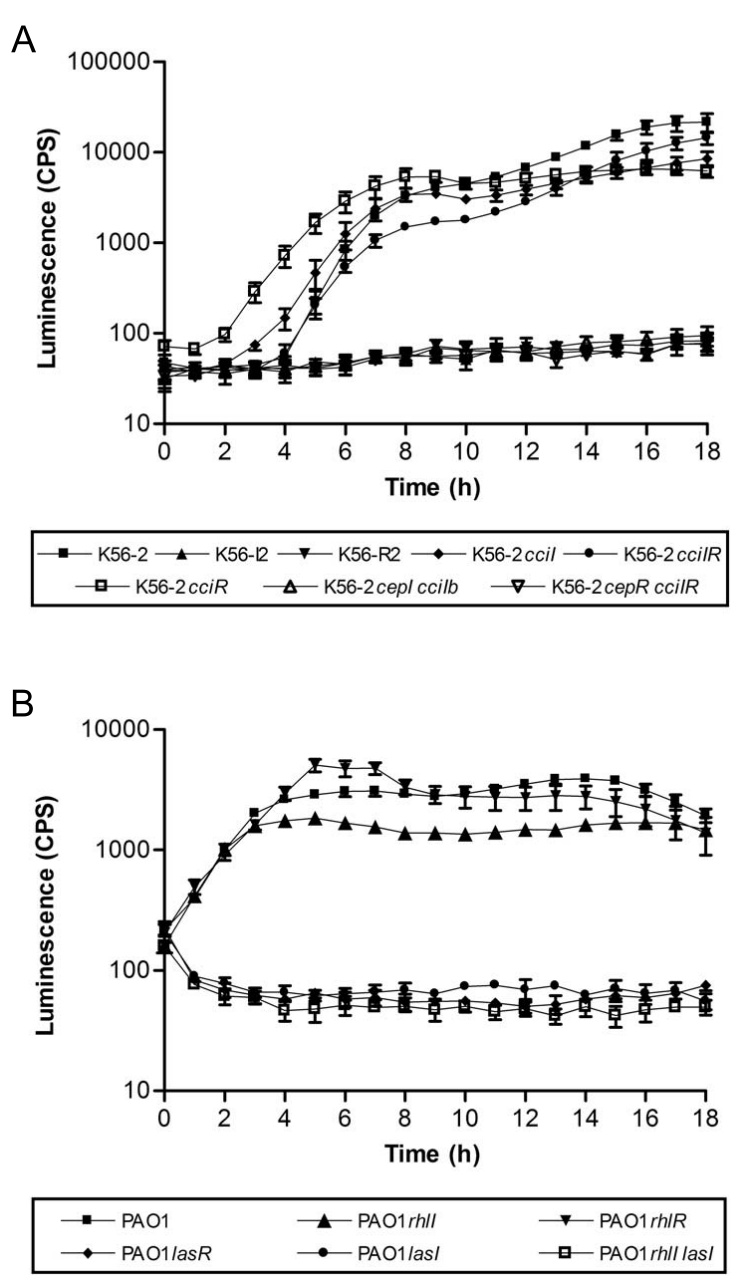
**Detection of AHL using the *A. tumefaciens *A136 (pCF218) (pMV26) as biosensor in liquid co-culture assay**. Detection and temporal expression of AHL in liquid co-culture assay for *B. cenocepacia *(A) and *P. aeruginosa *strains (B). Values shown are the mean ± standard deviation of at least four replicates.

In this study, we examined the utility of the *A. tumefaciens *A136 (pCF218) (pMV26) reporter system for semi-quantitative detection of AHLs during growth in co-culture with previously characterized QS mutants of *B. cenocepacia *and *P. aeruginosa *(Table 1). To monitor the production of AHLs over time, we co-cultured in liquid medium K56-2, PAO1, or their respective QS mutant strains (Table 1) with the *A. tumefaciens *A136 (pCF218) (pMV26) reporter strain and measured the luminescence over a period of 18 h (Fig. 1A and Fig. 1B). In parallel experiments, strains were grown in the absence of the reporter strain to confirm that the growth rates of the various QS mutants were not affected by the QS gene mutation (data not shown). The AHL expression levels of all *B. cenocepacia *QS mutant strains (Fig. 1A) are similar to those previously reported using thin-layer chromatography bioassay analysis [12,13]. In *B. cenocepacia *K56-2 no AHLs were detected when either *cepI *or *cepR *was mutated since CepR is essential for the expression of both the CepI/R and CciI/R QS systems [13]. Intermediate amounts of AHL were detected in *cciI *or *cciR *mutants. Interestingly, AHL production is detected earlier in growth in the *cciR *mutant. CciR negatively regulates *cepI *which likely accounts for the earlier expression of AHLs [13]. In *P. aeruginosa*, LasR is at the top of the hierarchy of both QS systems [17] and regulates expression of both *lasI *and *rhlI *synthases (Fig. 1B). Therefore *lasR *or *lasI *mutants have little detectable AHL, whereas the *rhlI *mutation results in a slight decrease in AHLs in the culture medium (Fig. 1B).

**Table 1:** Genotypes of bacterial strains used in this study.

Strain ^a^	Genotype	Reference or source
*B. cenocepacia*		
K56-2	Wild type	[21]
K56-I2	*cepI*	[11]
K56-dI2	*cepI*	[13]
K56-R2	*cepR*	[11]
K56-2*cciI*	*cciI*	[13]
K56-2*cciIR*	*cciI*, *cciR*	[13]
K56-2*cciR*	*cciR*	[13]
K56-2*cepI cciIb*	*cepI*, *cciI*	[13]
K56-2*cepR cciIR*	*cepR*, *cciI*, *cciR*	[13]
*P. aeruginosa*		
PAO1	Wild type	[22]
PAO1*rhlI *(PDO100)	*rhlI*	[23]
PAO1*rhlR *(PDO111)	*rhlR*	[23]
PAO1*lasR *(PAO-R1)	*lasR*	[24]
PAO1*lasI *(PAO214)	*lasI*	H. P. Schweizer
PAO1*rhlI lasI *(PAO-JP2)	*rhlI*, *lasI*	[25]

^a ^Names in parentheses are previously published strain names.

If this assay was employed with test strains with varying growth rates, the luminescence values could be normalized to optical density, although it would need to be noted that the normalized value would be the optical density of the combined cultures and not necessarily reflective of the cell density of the strain being analyzed for AHL production. The only strain shown in Fig. 1 with slower growth was PAO1*rhlIlasI *which doesn't produce AHLs regardless of growth phase. The strains shown in Fig. 1A and Fig. 1B reached similar OD 600 nm values in the co-culture assay suggesting that there were no growth affects due to co-culture.

We determined that K56-2 did not alter growth of the reporter strain by comparing the number of A136 present in co-culture or single culture at 0 and 24 hr. Cultures were plated on LB agar and *B. cepacia *selective agar (BCSA) [18]. The number of cfu recovered on BCSA was subtracted from the number of cfu recovered on L agar to determine the number of A136 present. There was no difference in the number of A136 present grown alone or with K56-2 and the ratio of A136 to K56-2 was similar at both timepoints (data not shown).

### Detection of AHLs using a soft agar co-culture assay

**Figure 2:**
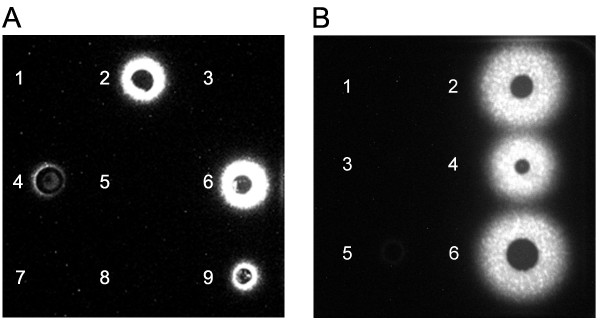
**Detection of AHLs using the *A. tumefaciens *A136 (pCF218) (pMV26) as biosensor on soft agar co-culture assay**. Detection of AHLs in a soft agar co-culture assay for *B. cenocepacia *(A) and *P. aeruginosa *strains (B). Panel A: 1, K56-2*cepI cciIb*; 2, K56-2*cciI*; 3, K56-2*cepR cciIR*; 4, K56-2*cciIR*; 5, K56-dI2; 6, K56-2*cciR*; 7, K56-I2; 8, K56-R2; 9, K56-2. Panel B: 1, PAO1*rhlI lasI*; 2, PAO1*rhlR*; 3, PAO1*lasI*; 4, PAO1*rhlI*; 5, PAO1*lasR*; 6, PAO1.

We previously developed a soft agar co-culture assay to detect AHL production in *B. cenocepacia *[19]. In this study, we determined if this assay could be used to qualitatively assess AHL production and if the results correlated with liquid co-culture assay. In this assay, the size of the AHL diffusion ring within the soft agar is directly proportional to the amount of AHL produced by the bacterial strain. When comparing the *B. cenocepacia *results on soft agar (Fig. 2A) to the results of the liquid co-culture assay (Fig. 1A), we observed that the non-producing AHL strains were negative in both methods. However, both *cciI *and *cciR *mutant strains produced more luminescence than the wild type K56-2 strain unlike in the liquid assay (Fig. 1A). It is possible that growth differences on the soft agar or regulatory pathways affected differently by these mutations on agar surfaces may explain the differences between the two assays for these strains. For *P. aeruginosa *strains, results from the soft agar assay could be extrapolated to the liquid co-culture assay (Fig. 2B).

### Application of the liquid co-culture assay as a high-throughput screening tool

**Figure 3:**
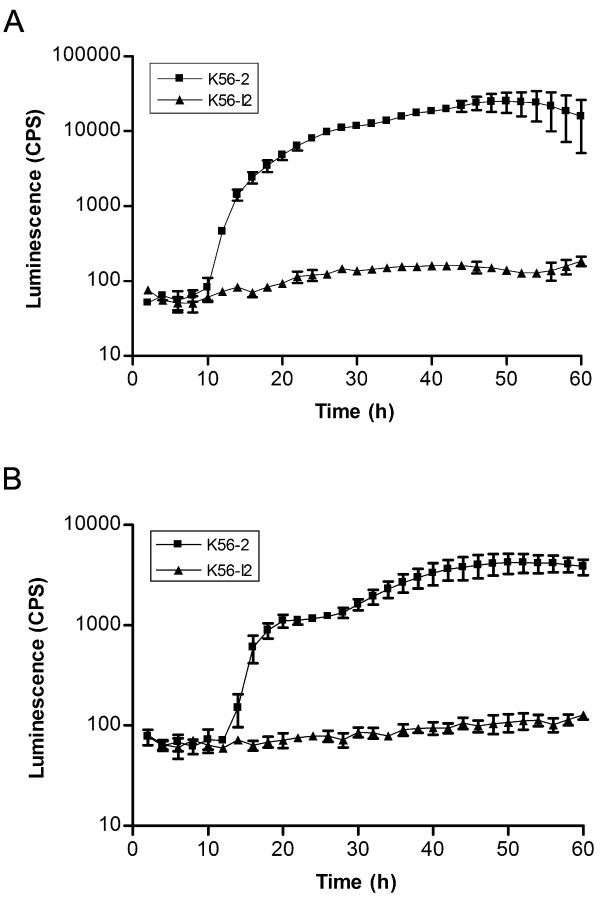
**Comparison of the detection of AHLs using the *A. tumefaciens *A136 (pCF218) (pMV26) as biosensor with different microtiter plate formats**. Temporal expression of AHL in liquid co-culture assay by *B. cenocepacia *strains in 96- (A) and 384-well formats (B). Values shown are the mean ± standard deviation of at least 3 replicates.

The liquid co-culturing system is adaptable for high-throughput screening. To demonstrate this we evaluated the assay using both a 96- and a 384-well plate format (Fig. 3) with *B. cenocepacia *strains K56-2 and K56-I2 (*cepI*) (Table 1). Although the CPS are lower in the 384-well format (Fig. 3B), due to reduced growth (data not shown), trends between the two formats were identical indicating that the 384-well plate format could also be utilized as a semi-quantitative assay.

## Conclusion

In summary, the co-culture assays described in both liquid and soft agar enable the detection and semi-quantitation of AHL production in *P. aeruginosa *and *B. cenocepacia *(Fig. 1 and Fig. 2) and should be applicable to AHL analysis in other bacterial species, providing the strains could grow in co-culture with the reporter strain. The reporter is sensitive to a wide range of AHLs [6]. However, the sensitivity of the luminescence counter and the camera system used is critical for the successful detection of a broad range of AHLs [6]. These two assays have enormous potential as high-throughput screening tools especially with the use of the 384-well format. Large libraries can quickly be screened and mutants defective in producing AHL easily identified by measuring CPS with the liquid assay. However, one of the most promising applications for this AHL detection assay might be for the identification of QS blockers by screening compound libraries in 384-well formats and looking for the absence of signal. Potential mutants or QS blockers identified using this screening assay could be validated using subsequent assays for the detection and measurement of specific AHLs, as well as assays that correlated AHL production to growth. With the increasing numbers of bacterial species resistant to all or most available antibiotics, alternative therapeutic approaches are needed. One strategy is to target bacterial virulence mechanisms [20]. Since QS systems control the expression of several virulence factors in many pathogens, compounds that block production of AHL signals or interfere with their activity may be effective anti-virulence agents.

## Methods

### Bacterial strains, plasmids and growth conditions

*A. tumefaciens *A136 (pCF218) (pMV26) [5,6] was grown in Luria-Bertani broth (LB) (Miller's broth base, Invitrogen, Burlington, Ontario, Canada) at 28 – 30°C for up to 24 h with the addition of 25 μg/ml of kanamycin and 4.5 μg/ml of tetracycline. *B. cenocepacia *and *P. aeruginosa *strains were grown in Trypticase soy broth (TSB; Becton, Dickinson and Company, Sparks, MD, USA) and LB respectively with antibiotics if required and incubated at 37°C overnight. Chemicals were purchased from Sigma-Aldrich Canada, Ltd., (Oakville, Ontario, Canada).

### Luminescence bioassays

For the detection and monitoring of AHL production, bioluminescence assays employing soft agar plates or liquid medium were developed using *A. tumefaciens *A136 (pCF218) (pMV26) as a reporter strain [5,6]. The soft agar co-culture assay was performed as previously described [19]. Briefly, an overnight culture of the reporter strain was mixed in a ratio 1:80 (v/v) with a mixture of TSB (Becton, Dickinson and Company) containing 0.7% agar (w/v). Each plate was prepared with 20 ml of the TSB agar reporter strain mixture and let dry for approximately 2 h at room temperature before use. Two μl of an overnight culture normalized to an OD_600 _of 0.3 of *B. cenocepacia *or *P. aeruginosa *were spotted onto the soft agar of the reporter plate, let dry for 20 min, and incubated at 28 – 30°C for 24 h. Pictures of the reporter plates were taken using a Fluorchem™ 8900 digital camera system (Alpha Innotech, San Leandro, California, USA) to detect luminescence. For the liquid co-culture assay, 150 μl or 75 μl of TSB (Becton, Dickinson and Company) or LB (Invitrogen) were added to each well of black, clear-bottom 96- and 384-well microtiter plates (Corning Inc., Corning, NY, USA), respectively. An overnight culture of the *A. tumefaciens *A136 (pCF218) (pMV26) reporter strain previously diluted 1:10 (v/v) was added to each well for a final dilution of 1:1500 (v/v). One μl of an overnight culture of *B. cenocepacia *K56-2 was added to each well containing the reporter strain. To prevent evaporation, 75 and 25 μl of mineral oil were added on top of each well of the 96- and 384-well plates, respectively. Plates were placed into a Wallac Victor2 Model 1420 Multi-label Counter (Perkin-Elmer Life Sciences, Boston, MA, USA), incubated at 28 – 30°C with constant shaking and luminescence was detected (CPS, counts per second) at hourly intervals. At least three replicates were performed for each assay.

## Authors' contributions

SPB developed the soft agar and the liquid co-culture assays, and drafted the manuscript. ALB helped develop the liquid co-culture assays. PAS supervised the study and contributed to the drafting of the manuscript. All authors have approved the final version of the manuscript.
